# Knowledge, Attitudes, and Management of General Practitioners of the Hospital Districts of Ouagadougou about Migraine (Burkina Faso)

**DOI:** 10.1155/2021/9327363

**Published:** 2021-11-18

**Authors:** A. A. Dabilgou, A. Dravé, J. M. A. Kyelem, A. Zoma, C. Napon, A. Millogo, K. Karfo, J. Kaboré

**Affiliations:** ^1^Department of Neurology, University Hospital Yalgado Ouedraogo, Ouagadougou, Burkina Faso; ^2^Department of Neurology, Regional University Hospital of Ouahigouya, Ouahigouya, Burkina Faso; ^3^Department of Neurology, University Hospital of Bogodogo, Ouagadougou, Burkina Faso; ^4^Department of Neurology, University Hospital of Sourou Sano, Bobo Dioulasso, Burkina Faso; ^5^Department of Psychiatry, University Hospital Yalgado Ouedraogo, Ouagadougou, Burkina Faso

## Abstract

**Background:**

Migraine is a common neurological disorder characterized by severe headache attacks that may be debilitating. The objective of this study is to determine the knowledge and attitudes of general practitioners in the hospital districts of the city of Ouagadougou on migraine.

**Methods:**

This cross-sectional study was carried out in hospital districts of Ouagadougou. The data were collected during three months from February 1 to April 30, 2020.

**Results:**

The study included 116 general practitioners. Thirteen percent of them were suffering from migraine. All participants had previous experience with migraine diagnosis before the survey. Eighty percent of general practitioners had a good level of knowledge of ICDH-3 criteria (knowing 6-7 criteria). The most widely recognized IHS criteria were pulsatility quality (93.1%), photophobia or sonophobia (80.2%), and mild-to-moderate intensity (80%). Ninety-five (81.9%) general practitioners rarely ordered brain imaging. The most common acute treatments were nonsteroidal inflammatory drug (39.47%), paracetamol (44.74%), and derivate of ergot (3.95%). The most common preventive treatments were amitriptyline (27.8%), derivate of ergot (18.9%), and NSAID (16.7%). The majority of general practitioners (56.9%) have referred headache patients to a colleague or specialist.

**Conclusions:**

Our study found that diagnostic criteria and acute treatment of migraine were well known by the majority of general practitioners, in contrast of preventive treatment.

## 1. Background

Migraine is a common neurological disorder characterized by severe headache attacks that may be debilitating [1, 2]. The diagnosis is based on the International Classification of Headache Disorders (ICHD-3 beta version) diagnostic criteria published in 2013. Migraine is one of the most common conditions presenting to both a primary care and general neurology practice [3]. The World Health Organization has declared migraine a major public health problem due to a lack of knowledge about its causes and effective treatment options [4]. Primary care is an important setting for the management of migraine, and in many countries, most migraine consultations occur in this context [5]. Migraineurs who consult PCPs for migraine may not receive a correct diagnosis [6] and/or may not receive adequate treatment [7]. There have been few studies investigating how to improve migraine education in the primary care setting [8–10]. There are African recommendations on the management of migraine in adults [11]. To our knowledge, there are not yet studies on the evaluation on the migraine management of GPs in Sub-Saharan Africa, as in Burkina Faso. The objective of our study was to assess the knowledge of general practitioners of the five hospital districts of Ouagadougou in order to optimize their knowledge, attitudes, and practices in the face of migraine.

## 2. Materials and Methods

### 2.1. Study Profile

We carried out a descriptive cross-sectional study in the five hospital districts of the city of Ouagadougou, during a period of 3 months from February 1 to April 30, 2020.

### 2.2. Study Population

The study population consisted of GPs of the five hospital districts of Ouagadougou who consented to participate in the survey.

### 2.3. Sampling

This was a systematic nonrandom sample of general practitioners working in the five hospital districts of the city of Ouagadougou.

### 2.4. The Questionnaire

Data were collected using a self-administered anonymous questionnaire comprising four parts: sociodemographic data of participants (age, sex, seniority, medical background, specific training on migraine, and internship in neurology); familiarity of migraine (number of migraineurs consulted per year and age of migraine patients at the time of diagnosis); migraine diagnosis (criteria of migraine, types of investigations in case of migraine, indication of these investigations, colleague opinion award, use of a headache diary, assessment of the handicap generated by migraine, and complications of migraine); and management of migraine (knowing VAS, type of acute treatment, conditions of initiation of acute treatment, conditions of initiation of prophylactic treatment, type of prophylactic treatment, and duration of prophylactic treatment). The questionnaire was semistructured in which the participant had to answer with <yes, <no, or <do not know or to choose one or more answers among those which were proposed to him. Some questions were open-ended. The initial version of the questionnaire was composed by three of the authors of this work (ZA, DAA, and CN) and was changed to the final form after revision for content validity and clarity by a pretest study that was carried randomly in 15 GPs working in other medical centers.

### 2.5. Data Collection and Analysis

#### 2.5.1. Procedure of Collection

GPs from the five district hospitals in the city of Ouagadougou were visited in the time of the survey. In each district, we first met with the district authorities to obtain lists of GPs and their consultation program. We met all the GPs and present them the authorization of Health Ministry of Burkina Faso. All the GPs presented in the time of the visit were asked to answer the questionnaires at their workplace. After receiving their verbal consent to participate, they received the paper version of the questionnaire. Depending on their availability, the questionnaire was completed in the time of the visit or later and returned back with care unit supervisors.

#### 2.5.2. Data Analysis

The data collected were analyzed by using Epi Info version 7.2. The quantitative variables were expressed as means and standard deviation. The qualitative variables were expressed in numbers and in percentage.

### 2.6. Assessments

We questioned GPs on the criteria which in the face of a headache make them pose the diagnosis of migraine without aura. They were evaluated according to knowing ICDH 3 diagnosis criteria of migraine without migraine. The level of knowledge was assessed into 4 levels: low level of knowledge: 0–4 criteria; adequate level of knowledge: 5 criteria; good level of knowledge: 6-7 criteria; and very good level of knowledge: all ICDH-3 criteria. Management of migraine was evaluated according to international guidelines. For acute treatment, the correct response was accepted if it contained the following: NSAIDs and/or aspirin and/or aspirin and metoclopramide and/or paracetamol or triptans. For the prophylactic treatment, the valid response was amitriptyline and/or oxetorone and/or pizotifen and/or propranolol and/or topiramate, valproate de sodium, and carbamazepine.

## 3. Results

### 3.1. Sociodemographic and Professional Characteristics

Our study population included 116 GPs of the 5 health districts of the city of Ouagadougou. The mean age of participants was 32 years. The majority of them were men (54.3%) and were graduated before 2015 (74.1%). The professional experience of the participants was 3.69 years. Forty-six GPs (39.6%) had already completed an internship in neurology during their initial medical training. All the GPs worked in public health centers. The socioprofessional data of GPs are given in Table 1.

### 3.2. Experience of Migraine

Sixteen participants declared themselves migraineur (13.8%) with a majority of women (56.2%). All participants had previous experience with migraine diagnosis before the survey. Of them, 95 (81.9%) reported having consulting 5–35 migraineurs during last year. For 81% of GPs, the mean age of migraineurs was between 20 and 30 years.

### 3.3. Migraine Diagnosis

Table 2 shows the criteria used by the GPs to establish the diagnosis of migraine without aura (*N* = 116). Twenty-three GPs (19.8%) had very low knowledge of ICDH-3 criteria of migraine without aura and 93 (80.2%) had a good level of knowledge of ICDH-3 criteria. The most widely recognized IHS criteria were pulsatility quality (93.1%), photophobia or sonophobia (80.2%), and moderate or severe intensity (80%). At least sixty-seven (57.8%) GPs gave a wrong answer. The wrong responses were bilateral location (53.5%), very severe form of headache (19.8%), sudden onset (6.9%), and headache in helmet (5.2%). According to investigations in case of migraine, 95 (81.9%) GPs rarely ordered brain imaging and 21 (18.1%) regularly or systematically prescribed brain imaging. The patients who have benefited from the brain scan were those presenting an abnormal neurological examination (67.2%), a migraine after 50 years (63.79%), an atypical aura (51.72%), and an attack duration over 4 hours (2.6%) and over 7 days (18.10%). The other investigations prescribed by GPs were an ophthalmologic exploration (56.03%), an electroencephalogram (37.07%), and a sinus X-ray (18.10%).

### 3.4. Migraine Management

Sixty-nine participants (59.48%) answered the question regarding visual analog scale for pain. Of these, 49 (86.95%) gave a correct definition of visual analog scale for pain while 20 (17.24%) reported using it in routine practice. The strategies of acute treatment were based on VAS for pain (90.5%) and repercussion on daily life (97.4%).  Table 3 gives the drugs used by GPs in the management of acute and chronic migraine. Of 115 GPs who answered the question relative to medications of migraine acute treatment, 60 (39.47%) said they would use an NSAID, 68 (44.74%) paracetamol, and 3.95% derivate of ergot. The proportion of correct answers was 98%. All the participants had answered the question concerning the initiation of prophylactic treatment of migraine. The majority of them thought that migraine prophylactic treatment should be started when the acute treatment has failed (66.4%), according to the impact on daily life (63.8%), frequency of attacks (62.9%), when patient had more than 3–6 episodes of acute migraine per month (44.8%), and intensity of headache on VAS for pain (25%). A total of 90 (77.6%) GPs answered the question according to prescription of prophylactic treatment. Twenty-five respondents (27.8%) would use amitriptyline, 25 (18.9%) would use derivate of ergot, and 15 (16.7%) would use NSAID. Forty percent of respondents would use recommended molecule. Of the 114 respondents, 43 (37.7%) GPs regularly assessed the effectiveness of the prophylactic treatment of migraine. This evaluation took place one month (55.8%), 3 months (39.5%), and 6 months after initiation of prophylactic treatment (4.6%). One hundred and eleven GPs answered the question on the duration and the effectiveness of prophylactic treatment. Of these, 18 (16.2%) judged effectiveness after reducing headache frequency by 25%, 69 (62.2%) after 50% reduction in headache frequency, and 24 (21.6%) after 75% reduction in headache frequency. When the prophylactic treatment was effective, that was stopped 3 months after its initiation (12.9%), 6 months after (40.5%), 12 months after (6%), or never (26.7%).

### 3.5. Referral Pattern

The majority of general practitioners (56.9%) have referred headache patients to a colleague or specialist. The main reasons for referral were better treatment (69.7%), systematically (25.75%), and for diagnosis (4.54%). The full version of study questionnaire is given in Table 4.

## 4. Discussion

This cross-sectional study is the first kind in Burkina Faso, a developing country in West Africa to determine the knowledge, attitudes, and practices of GPs working in the primary health level about migraine. Patients received the right diagnosis only just at the lowest level in primary health care services [12, 13]. The majority of GPs in our study (80.2%) had a good level of knowledge of ICDH criteria of migraine without aura. This high level of knowledge could be explained by the familiarity of GPs toward migraine. Most GPs had experienced migraine attacks (13.8%), in high proportion than those in general population (5.6%) [14]. Otherwise, healthcare professionals (HCPs) have stressful jobs, are frequently on rotating work shifts, undergo emotional stress, and work long hours every day because of their job requirements [15, 16]. This high prevalence could be explained by the fact GPs with migraine may be more interested in the study than other doctors. Similar proportion was observed for primary health care physicians in Turkey (15%) [17], but higher proportion for GPs was observed in Spain (27.4%) [18] and in Germany (24.6%) [19]. All the GPs in our study had already made a migraine diagnosis before the survey, in contrast with the study of Gültekin (8.3%) [17]. This situation could be explained by the fact that a great number of them (39.6%) had already completed an internship in neurology during their initial medical training. The vast majority of GPs (81.9%) had already reported having received 5–35 migraineurs last year. This frequency seems lower compared to that encountered in other countries such as Pakistan, where among 449 family physicians, 15.1% of them had reported seeing 21–50 migraine patients per month [20]. The most common diagnostic criteria knew by GPs were pulsatile quality (93.1%), presence of photophobia or phonophobia (80.2%), mild or severe form of headache (80%), and increase in headache with physical activity (79.3%). These results were different with the study of Gültekin which found unilateral headache (53%), throbbing characteristic (47%), presence of nausea or vomiting (45.5%), and presence of photophobia or phonophobia (41.7%) [17]. Regarding paraclinical explorations, studies have shown that primary care GPs have a great tendency to prescribe radiological investigations in the event of chronic headache [21, 22]. Regarding investigations, 81.9% of GPs prescribed brain scan on a case-by-case basis, in the event of abnormal clinical examination (67.2%), migraine attacks appearing after the age of 50 years (63.8%) and atypical aura (51.72%). All these results were in line with revised French guidelines [23]. However, some requests were unjustified, namely, in cases of migraine attacks lasting 4 hours (18.1%) and those dating more than 7 days (2.6%). The brain scan was systematically requested in 18.1%, despite the recommendations [23]. A study from Turkey found that 33.1% of PHCP had expressed the view that laboratory inquiry and brain screening are necessary for migraine diagnosis [17]. Other investigations such as an ophthalmologic exploration (56%), an electroencephalogram (37.1%), and a sinus X-ray (18.1%) are not justified because there is no indication to perform these investigations in a patient with migraine defined according to IHS criteria (professional agreement) [23]. The most common acute treatments were paracetamol (44.7%), NSAID (39.5%), and derivate of ergot (4%). In Pakistan, 50.9% of physicians identified NSAIDs before triptans (34.6%), as being the most common effective treatment for acute migraine [20]. According to MacGregor, a significant proportion of PCPs prescribe simple analgesics and nonsteroidal anti-inflammatory drugs (NSAIDs) for migraine (even for severe attacks), rather than migraine-specific treatments [7]. On the other hand, in industrialized countries such as France, more than 75% of GPs prescribed triptans as part of the treatment of migraine attacks [24]. Migraine-specific treatments, triptans (2.6%) and ergotamine derivatives (4%), were less prescribed by GPs in our context because of their unavailability and their high cost. According to prophylactic treatment, the majority of GPs thought that it should be started when the acute treatment has failed (66.4%), worse impact on daily life (63.8%), and increase of migraine attacks (62.9%). These conditions correspond to the recommendations of the African guideline [11]. However, GPs in comparison with neurologists tend to institute prophylactic treatment for lower thresholds [25]. Twenty-two percent of GPs did not answer the question regarding the type of medications they can use for prophylactic treatment, for fear of giving wrong answer. Among the respondents, 39.8% of them had used recommended preventive medications, namely, amitriptyline (27.8%), topiramate (13.3%), and propranolol (8.9%). Pharmacologic agents with the most clinical experience in chronic migraine (CM) include beta-blockers, topiramate, and amitriptyline [26–29]. In our series, two GPs prescribed molecules reserved for the prophylactic treatment in the treatment of migraine attacks, namely, propranolol and amitriptyline. This trend was also observed in Pakistan where 19% of physicians prescribed topiramate in the treatment of migraine attacks [19]. Obviously, this was a therapeutic error that could worsen the migraine attack or the need to train doctors on the issue. Conversely, several drugs for the treatment of the migraine attack were prescribed in the prophylactic treatment of migraine, namely, derivate of ergot (18.9%), NSAID (16.7%), tramadol (2.2%), and paracetamol (4.4%). Acute treatments are allowed for patients who are having frequent and severe migraine episodes [30]. Only 38% of respondents regularly assessed the effectiveness of prophylactic treatment. This low percentage could be explained by the fact that most patients no longer consult after the improvement of the seizures. Thirty-nine percent of respondents prescribed monitoring of the efficacy of the prophylactic treatment of migraine within the time limit (at 3 months), in accordance with African recommendations, while the majority of respondents (55.8%) did so at one month early [12]. The majority of respondents (62.2%) judged the effectiveness after a 50% reduction in the frequency of seizures, in accordance with the literature [31]. The majority of respondents stopped the prophylactic treatment of migraine before 8 months (53.4%), 6% after 12 months, and 26.7% never stopped the prophylactic treatment of migraine. This attitude is at odds with the data in the literature which states that the discontinuation of the prophylactic treatment should be done after 8–12 months [31]. According to referral pattern, 56.9% of GPs would refer the patients to another GP or specialist. Of them, 69.7% would refer the patients for the treatment of the migraine, in higher proportion than in a study from India which states that 5% of general physicians were referring the patients to the neurologist for the treatment [32]. However, the most common reason for referral was management of migraine (69.7%), in contrast with the study of Fearon in which diagnostic clarification (53.5%) was the most common reason [33].

## 5. Limitations of the Study

This cross-sectional study had some limitations. Indeed, the anonymity of the data collection sheet did not allow us to differentiate the respondents according to their place of practice with a view to a possible comparison. In addition, the principle of self-assessment remains subjective, influenced by the desire to answer correctly. Finally, we could think that only practitioners interested in the subject have answered this questionnaire based on voluntary service. As the collection period coincided with the presence of coronavirus disease, many GPs remained inaccessible and some health centers were closed for a while. This notwithstanding the survey being declarative, with open and closed questions, was an advantage. In view of the number of responses, this was a common pathology with a marked interest in GPs.

## 6. Conclusion

Our study found that diagnostic criteria and acute treatment of migraine were well known by the majority of general practitioners, in contrast to preventive treatment. It is important to establish continuous training for GPs in order to optimize their knowledge, attitudes, and practices in the face of migraine.

## Figures and Tables

**Table 1:** Sociodemographic and professional characteristics of GPs (*N* = 116).

Variable	Study population (*N* = 116)	Percentage
Age group (years)		
< 30	41	35.3
30–40	72	62.1
> 40	2	2.6
Male gender	63	54.3
History of migraine	16	13.8
Professional experience (years)		
1–5	86	74.1
5–10	25	21.6
>10	5	4.3
Completed internship in neurology	46	39.6
Number of migraineurs received last year	50	43.1

**Table 2:** Criteria used to establish the diagnosis of migraine (*N* = 116).

Diagnostic criteria for migraine	Study population (*N* = 116)	Percentage
Pulsating quality	108	93.1
Moderate or severe form of headache	93	80
Presence of photophobia or phonophobia	93	80.2
Increase in headache with physical activity	92	79.3
Bilateral headache	67	57.8
Presence of nausea or vomiting	62	53.4
Pain lasting for about 4–72 hours	60	51.7
Unilateral headache	47	40
Severe or very severe form of headache	23	19.8
Sudden onset	8	6.9
Headache in helmet	6	5.2

**Table 3:** Drugs used by GPs in the management of acute and chronic migraine.

Variable	Number (*n* = 115)	Percentage
Drugs used in the management of migraine (*n* = 115)		
Paracetamol	56	48.7
NSAID	46	40
Caféine	6	5.2
Derivate of ergot	5	4.3
Triptan	3	2.6
Tramadol	2	1.7
Amitriptyline	1	0.9
Propranolol	1	0.9
Drugs used in the management of chronic migraine (*n* = 90)		
Amitriptyline	25	27.8
Derivate of ergot	17	18.9
NSAID	15	16.7
Topiramate	12	13.3
Triptan	11	12.2
Propranolol	8	8.9
Ergotamine, caféine, and paracetamol	6	6.7
Paracetamol	4	4.4
Tramadol	2	2.2
Paracetamol and codeine	2	2.2
Paracetamol, caféine, and codeine	2	2.2

**Table 4:** Full version of the questionnaire.

Variable	Yes	No
Gender		
Male		
Female		
How old are you (years)?		
<30		
30–40		
≥40		
Years of experience		
Experience of migraine		
Number of migraineurs received last year		
Are you completed internship in neurology?		
How old are the patients at the time of migraine diagnosis?		
Give the criteria used to establish the diagnosis of migraine		
Pulsatile		
Brutal		
Mild		
Moderate or severe		
Severe or very severe		
Duration 4–72 h		
Lasts 30 min-7 days		
Unilateral		
Bilateral		
Retro-orbital and temporal		
In helmet		
Nausea/vomiting		
Photophobia/phonophobia		
Worsened by routine physical activity		
Do you order an additional examination in a migraine patient?		
If so, under what condition?		
All migraine patients		
Inaugural migraine after 50 ans		
Migraine with atypical aura (sudden onset or lasting more than an hour)		
Throbbing pain always on the same side		
Abnormal clinical examination		
Migraine lasting more than 4 hours		
Migraine lasting more than 7 days		
What type of investigation do you prescribe?		
Sinus X-ray		
Brain scan		
Brain MRI		
Blood ionogram		
EEG		
Ophthalmologic examination		
Other(s) to be specified/radio des sinus		
Have you ever taken the advice of colleagues in front of a migraine patient?		
If so, from whom? (many possible responses)		
Neurologist		
General practitioner		
Psychiatrist		
Other(s) to be specified		
What are the reasons for referring these patients with migraine?		
For better diagnostics		
Therapeutic problems		
Systematically		
Other reasons:		
Do you use a migraine diary?		
Do you assess the handicap caused by migraine in your patients?		
If yes, how?		
Feelings expressed by the patient?		
Number of migraine attacks?		
Frequency of consultations for this reason		
Impact on quality of life		
Give some migraine complications		
Do you use the VAS to assess the pain of your patients		
Based on what criteria do you institute a treatment for a migraine attack?		
Impact on daily life		
Worsening pain on VAS		
List the molecules used in the treatment of chronic migraine		
Name molecules used in the treatment of migraine attacks		
What are the criteria for establishing a long-term treatment for chronic migraine?		
VAS pain intensity		
Failure of first-line treatment		
Impact on daily life		
Frequency of seizures		
If so, how often?		
From what decrease in the frequency of seizures is the treatment of chronic migraine effective?		
25%		
50%		
75%		
Do you carry out an evaluation of the effectiveness of the treatment of chronic migraine?		
If so, how long after starting the treatment of chronic migraine?		
3 months		
6 months		
9 months		
The long-term treatment started being effective, you stop it after how long?		
Never		
3 months		
6 months		
12 months		
>1 year		

## Data Availability

The data used to support the findings of this study are available from the corresponding author upon request.

## Abbreviations

PCPs: Primary care professionals
HCPs: Healthcare providers
PHCP: Primary healthcare providers
CM: Chronic migraine
ICHD-3: International Classification of Headache Disorders
NSAIDs: Nonsteroidal anti-inflammatory drugs
IHS: International Headache Society
VAS: Visual analog scale
GPs: General practitioners.
